# Pigmented villonodular synovitis of the hip in systemic lupus erythematosus: a case report

**DOI:** 10.1186/1752-1947-5-443

**Published:** 2011-09-07

**Authors:** Hans-Joachim Anders

**Affiliations:** 1 Department of Nephrology, Medizinische Poliklinik, University of Munich, Munich, Germany

## Abstract

**Introduction:**

Pigmented villonodular synovitis is a rare disease of unknown etiology mostly affecting the knee and foot. Until now an association with autoimmune diseases has not been reported.

**Case presentation:**

The diagnosis of systemic lupus erythematosus was made in a 15-year-old Caucasian girl based on otherwise unexplained fatigue, arthralgia, tenosynovitis, leukopenia, low platelets and the presence of antinuclear and deoxyribonucleic antibodies. At the age of 20 a renal biopsy revealed lupus nephritis class IV and she went into complete remission with mycophenolate mofetil and steroids. She was kept on mycophenolate mofetil for maintenance therapy. At the age of 24 she experienced a flare-up of lupus nephritis with nephrotic syndrome and new onset of pain in her right hip. Magnetic resonance imaging, arthroscopy and subtotal synovectomy identified pigmented villonodular synovitis as the underlying diagnosis. Although her systemic lupus erythematosus went into remission with another course of steroids and higher doses of mycophenolate mofetil, the pigmented villonodular synovitis persisted and she had to undergo open synovectomy to control her symptoms.

**Conclusion:**

Systemic lupus erythematosus is associated with many different musculoskeletal manifestations including synovitis and arthritis. Pigmented villonodular synovitis has not previously been reported in association with systemic lupus erythematosus, but as its etiology is still unknown, the present case raises the question about a causal relationship between systemic lupus erythematosus and pigmented villonodular synovitis.

## Introduction

Pigmented villonodular synovitis (PVNS) is a rare monoarticular proliferative synovial disorder of unknown etiology mostly affecting the knee, foot or the hip [1]. Metastatic disease was not observed in large cases series, therefore PVNS is considered to represent a benign synovial tumor [2]. However, the fibrocellular nature of PVNS tissue can cause pain, disability and progressive destruction of cartilage and bone, especially when the hips are affected [1-5]. The male to female ratio of patients with PVNS is around 2:3 [1,2]. Diffuse forms of PVNS in large joints frequently relapse even after synovectomy [6].

Systemic lupus erythematosus (SLE) is a rare autoimmune disorder directed against ubiquitous nuclear autoantigens, immune complex disease and various forms of organ inflammation [7]. The male to female ratio of SLE patients is 1:9 [7]. Musculoskeletal manifestations of SLE include arthralgia, myalgia, myositis, and rarely synovitis, although periarticular and destructive ligamental inflammation can occur. Although both diseases are most prevalent in adolescents a rigorous PubMed/Medline search did not reveal any previous report about PVNS in SLE.

## Case presentation

A previously healthy 15-year-old Caucasian girl with Italian-German parents presented with new onset of fatigue, diffuse arthralgia, butterfly rash, tenosynovitis of the wrist, lymphopenia and thrombocytopenia. In the absence of other explanations and the presence of antinuclear antibodies (ANA, 1:7680, granular pattern), anti-double stranded deoxyribonucleic acid (dsDNA) antibodies (69 U/mL) and hypocomplementemia the diagnosis of SLE was made. All symptoms resolved with 130 mg prednisolone followed by dose-tapering and azathioprine at a dose of 100 mg/d.

At the age of 20 acute appendicitis (treated by open surgery) was followed by persistent high fever and rashes, fatigue, diffuse arthralgia, leucopenia and hypocomplementemia. A lupus flare-up was suspected. ANAs and anti-dsDNA were 1:7680 and 2713 U/mL, respectively. The prednisolone maintenance dose of 5 mg/d was increased to 1 mg/kg body weight along with 100 mg azathioprine. However, because of 2.5 g proteinuria over 24 hours and dysmorphic erythrocyturia (serum creatinine 1.1 mg/dL), a renal biopsy was performed and displayed diffuse proliferative lupus nephritis (class IV). Our patient received six 500 mg pulses of cyclophosphamide according to the Euro-Lupus protocol [8] and was subsequently treated with 2 g/d mycophenolate mofetil as a maintenance therapy. Complete remission of proteinuria was reached 18 months after initiation of this regimen so the low dose prednisolone was stopped.

**Figure 1:**
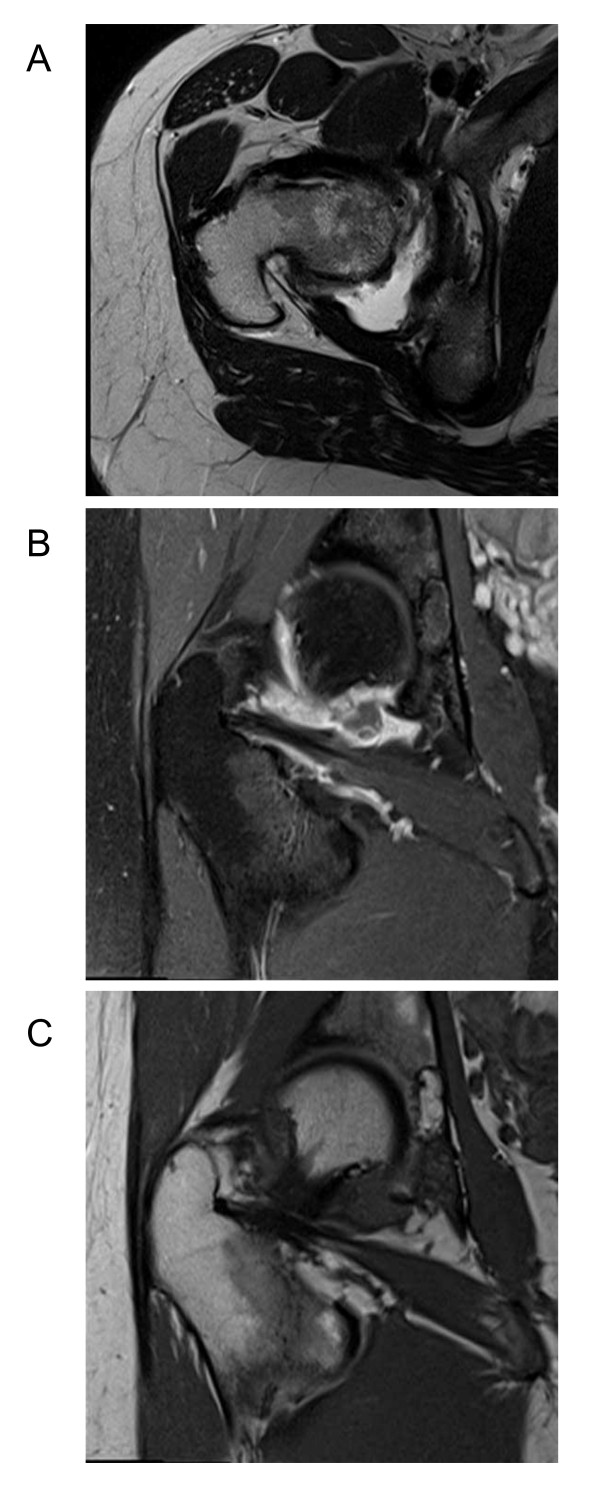
**MRI of her right hip joint**. (A) The T1-weighted coronal image illustrates the synovial fluid effusion (white) in the dorsal recessus of the joint around the femoral head. (B) Gadolinium contrast of sagittal images shows diffuse enhancement in synovial tissue along the zona orbicularis to the posterior joint cavity surrounding a contrast-free corpus librum of 4 mm diameter. (C) The synovial proliferation appears in dark grey in the T2-weighted image at the same location. Bone or cartilage did not display erosions or thinning, respectively.

Three years later after stepwise reduction of the mycophenolate mofetil down to 1 g/d our patient developed pain in her right hip, lymphopenia, hypocomplementemia, erythrocyturia and massive proteinuria of 10 g/d. A flare-up of SLE and lupus nephritis was suspected. She was put on prednisolone 1 mg/kg body weight and the dose of mycophenolate mofetil was increased to 2 g/d, and later to 3 g/d. She was also put on chloroquine but stopped it shortly after because of new-onset of alopecia. Partial improvement of her proteinuria was reached one year later (200 mg/d). Since then her SLE-related symptoms and laboratory parameters have remained stable suggesting sustained remission. Only her hip pain persisted and had not at all responded to the high doses of prednisolone. Magnetic resonance imaging (MRI) of her right hip suggested the diagnosis of villonodular synovitis and subsequent arthroscopy of her right hip and subtotal synovectomy confirmed the diagnosis of PVNS. Her hip pain resolved but reoccurred two years later when another MRI indicated remittent PVNS without evidence of osteoarthritis, arthritis, or osteonecrosis (Figure 1). Plain X-rays were normal. Our patient underwent rigorous synovectomy by open surgery, which subsequently controlled all PVNS-related symptoms.

## Discussion

A rigorous PubMed/Medline research did not reveal any previous reports about an association between PVNS and the key words "lupus", autoimmunity", "kidney" or "proteinuria", rendering a causal relationship between the underlying SLE or lupus nephritis and PVNS to be unlikely. It is of note that the reports on larger series of PVNS mostly lack a detailed description of comorbidities. However, in our patient the symptoms of PVNS clearly developed in a temporal association with a flare-up of SLE and lupus nephritis. We considered that pigmented synovitis could be secondary to chloroquine treatment which often causes hyperpigmentation of the skin and mucus membranes. However, our patient had not been exposed to antimalarial drugs before the PVNS diagnosis was made and an association between PVNS and chloroquine treatment has also not been reported. PVNS is almost equally prevalent in males and females while SLE has a 1:9 male to female ratio, which also argues against a shared pathogenesis. This includes a potential role of estrogens which clearly contribute to onset and disease activity of SLE while an association of estrogens and PVNS remains speculative [9]. Furthermore, SLE remains a recurrent disease with flares of synovitis while open synovectomy can result in persistent cure of PVNS [2-5]. As the precise cause of PVNS to date remains unknown it might still be worthwhile to consider that either the pathomechanisms that drive SLE disease activity or its consequences on tissue homeostasis have an impact on the factors that drive PVNS. For example, a study that compared histopathological characteristics of synovitis in rheumatoid arthritis and diffuse PVNS found an overlapping pattern of proliferating macrophages and fibroblasts [10]. CD68/CD163+ synoviocytes were preferentially located in the vicinity of the synovial lining layer of rheumatoid arthritis patients while they were randomly distributed in PVNS [10]. In addition, 20% of synoviocytes were aneuploid in diffuse PVNS while all samples of focal PVNS or rheumatoid arthritis were diploid [10]. It will depend on future reports to see whether PVNS and SLE represent an accidental coincidence in our case or whether there is an association between these two disorders that has not been previously recognized. Monoarticular arthralgia not responding to immunosuppressive therapy in lupus patients should raise suspicion of alternative diagnoses such as PVNS.

## Conclusion

This is the first reported association between PVNS and SLE which might simply represent an accidental coincidence of two rare diseases or indicate that they share triggers for synovial overgrowth.

## Consent

Written informed consent was obtained from the patient at adult age for publication of this case report and any accompanying images. A copy of the written consent is available for review by the Editor-in-Chief of this journal.

## Competing interests

The authors declare that they have no competing interests.
